# Supplementary material for: Larger but younger fish when growth outpaces mortality in heated ecosystem
Source: eLife. 2023 May 9;12:e82996. doi: 10.7554/eLife.82996 (PMC10168697; doi:10.7554/eLife.82996)
Supplement: Supplementary file 1. — (a) Comparison of von Bertalanffy growth models with different combinations of shared and area-specific parameters (ordered by the difference in expected log pointwise density (elpd) from the best model). Note that in all models, L∞j and Kj vary among cohorts. (b) Comparison of allometric growth models with common or unique θ-parameter (exponent in the allometric growth model), ordered by the difference in expected log pointwise density (elpd) from the best model. [file elife-82996-supp1.docx]

Supplementary file 1a.

| Model Name | Model structure | elpd_diff |
| --- | --- | --- |
| M1 | Area-specific ${L_{\infty}}_{j}$, $K_{j}$ and $t_{0}$ | 0 |
| M4 | Area-specific ${L_{\infty}}_{j}$ and $K_{j}$, common $t_{0}$ | -9.8 |
| M2 | Area-specific $K_{j}$, common $t_{0}$ and ${L_{\infty}}_{j}$ | -112 |
| M3 | Area-specific $t_{0}$ and ${L_{\infty}}_{j}$, common $K_{j}$ | -152 |
| M7 | Area-specific ${L_{\infty}}_{j}$, common $K_{j}$ and $t_{0}$ | -158 |
| M6 | Area-specific $K_{j}$, common $t_{0}$ and ${L_{\infty}}_{j}$ | -175 |
| M5* | Area-specific $t_{0}$, common $K_{j}$ and ${L_{\infty}}_{j}$ | -1338 |
| M8* | Common $t_{0}$, $K_{j}$ and ${L_{\infty}}_{j}$ | -2155 |

* Models did not converge

Supplementary file 1b. Comparison of allometric growth models with common or unique $\theta$-parameter (exponent in the allometric growth model), ordered by difference in expected log pointwise density (elpd) from the best model.

| Model Name | Model structure | elpd_diff |
| --- | --- | --- |
| M1 | Intercept ($\alpha_{j\left[ i \right],k[i]}$) varying across individuals within cohorts, fixed, area-specific slope ($\theta_{ref}, \theta_{heat})$ | 0 |
| M2 | Intercept ($\alpha_{j\left[ i \right],k[i]}$) varying across individuals within cohorts, “fixed” common slope ($\theta)$ | -1.9 |
